# Psychiatry

**Published:** 1905-01-14

**Authors:** 


### PSYCHIATRY.

(*Continued from page 267.*)

**Katatonic Dementia.**—This subject is discussed by Mouratoff[2] who asks whether the katatonic form of dementia observed in young adults is a morbid entity or whether it is a clinical "symptoms-complex" which might enter into the most varied psychic troubles. The opinions of authors may be divided into three groups; some of the authors do not regard katatonia as independent. They state that it is met with in diverse mental disorders, as a complication of one or another typical mental malady. A second set recognise the complete independence of katatonia. The third set of authors admit the independence of katatonia, but interpret its symptoms and trace its cardinal manifestations differently. Kahlbaum, Schüle, and Kraepelin regard katatonic dementia as a grave degenerative psychosis, essentially with a basis of dementia on which are superposed the characteristic symptoms of waxy rigidity of the limbs (flexibilitas cerea), habitual stereotypic poses, negativism, verbigeration, automatic consciousness, and immobility for shorter or longer periods. Schüle and Kraepelin insist upon the morbid heredity of katatonics. Mouratoff agrees with those who attribute katatonia etiologically to a degenerate nervous heritage. This is found from the study of personal and family histories of patients. There is, however, ground for believing that while the dementia of katatonia is the essential feature, the katatonic symptoms themselves are the outcome chiefly of some obscure condition of toxæmia to which such dementia is prone. So that while katatonia is closely associated—especially by Schüle and Kraepelin—with the group of "degenerative psychoses," or "psychic degeneracies," it is very probable that the special lesion of katatonia is prone to act and develop only in certain groups of such degeneracies to the exclusion of others. Thus, according to von Grabe,[3] "katatonic symptoms occurring in true paranoia must be looked upon as only an episode." He relates the case of a female, 38 years old, in whom, since the age of 34 years, the katatonic symptom-complex developed, which after having persisted for four or five months, gradually gave way to the former delusional condition. Discussing the features of this case, von Grabe concludes that the delusions were too well systematised, and the history too characteristic for it to be considered as one of the paranoid forms of dementia præcox, and that the katatonic symptoms must be looked upon as being only an episode in a case of the paranoias. Fuchs[4] gives the interesting history of a case of a young man who developed an acute attack of insanity with katatonic symptoms. This passed away, but for the next ten years the patient presented the typical picture of development of paranoia, with systematised delusions of persecution and of grandeur, and with little or no mental weakness. A new exacerbation with hallucinations, illusions, and typical katatonic symptoms then occurred, lasting six months. After this period the patient became quieter, but his mental condition did not clear up this time, and he deteriorated in physical health and died. The autopsy is reported to have shown nothing characteristic. In the case, however, of the autopsy of a patient who had died of dementia præcox (a woman in whom the mental symptoms began at the age of 37 years), and who died from marasmus and pneumonia at the age of 43, W. R. Dunton reports,[5] in a very complete account, that changes were found in various organs, but were not characteristic of the disease. In the nervous system the meninges showed chronic cell proliferation. Cell preparations made from a number of regions of the cerebral and cerebellar cortex, and from the spinal cord, showed increased accumulation of pigment in the cell-bodies, chromatolysis generally central, atrophy and displacement of the nuclei, with slight increase of neuroglia nuclei round about the nerve-cells. The Weigert-Pal method seemed to show that the tangential nerve-fibres of the cortex were decreased in number. It could not be definitely ascertained whether the neuroglia fibres were decreased or increased in quantity. From his findings and those of other investigators, Dunton thinks that in dementia præcox we have a degenerative psychosis probably of autotoxic origin.

[2] Archives de Neurol., Jan., 1904. [3] Allgem. Zeitsch. für Psychiatrie, Part I., 1904. [4] Ibid., Part III., 1904. [5] Amer. Jour. of Ins., April, 1904.

(*To be continued.*)

